# Effects of Doenjang, a Traditional Korean Soybean Paste, with High-Salt Diet on Blood Pressure in Sprague–Dawley Rats

**DOI:** 10.3390/nu11112745

**Published:** 2019-11-12

**Authors:** Eun-Gyung Mun, Jung Eun Park, Youn-Soo Cha

**Affiliations:** Department of Food Science and Human Nutrition, Jeonbuk National University, 567 Baekje-daero, Deokjin-gu, Jeonju-si, Jeollabuk-do 54896, Korea; egmun1982@gmail.com (E.-G.M.);

**Keywords:** high-salt diet, blood pressure, doenjang, renin, soybean paste

## Abstract

Fermented foods in Korea contain a lot of salt. Although salt is reported to exacerbate health trouble, fermented foods have beneficial effects. We hypothesized that doenjang could reduce blood pressure in Sprague–Dawley (SD) rats fed a high-salt diet. Eighteen SD rats were divided into three groups: normal-salt (NS) group, high-salt (HS) group, and high-salt with doenjang (HSD) group. The salinity of doenjang and saltwater was adjusted to 8% using Mohr’s method. Blood pressure was significantly reduced in the HSD group compared with the HS group. Water intake and urine excretion volume has significantly increased in the HS group compared with the HSD group. The excreted concentrations of urine sodium, urine potassium, and feces potassium significantly increased in the HSD group compared with the HS and NS groups. Renin level was significantly decreased in the HSD group compared to the other groups. These results indicate that eating traditional salty fermented food is not a direct cause of hypertension, and the intake of doenjang in normal healthy animals improved blood pressure.

## 1. Introduction

Doenjang, Korean soy paste, is a fermented soybean product in traditional Korean cuisine. Doenjang is the basis of various dishes such as soup, stew, and salad dressing. Doenjang is produced by fermentation with *Bacillus subtilis* and mold such as *Aspergillus*, *Rhozopus*, and *Mucor* species. Doenjang has a long fermentation period that extends from 2 to 24 months. Due to its fermentation, doenjang contains a high level of bioactive compounds such as isoflavones and saponins [1]. In addition, doenjang is a good source of essential amino acids, minerals, vitamins, and phenolic compounds [2,3].

In previous studies, doenjang showed anti-obesity effects in rat fed with high-fat diet [4], and overweight adults [5,6,7], anti-diabetic effects in high-fat diet-induced obese mice [8,9], anti-cancer effects in carcinogenic mice [10,11], and anti-inflammation effects in mice [12,13,14]. The intake of doenjang reduces body fat mass in overweight subjects [5] and decreases visceral or abdominal fat mass in overweight subjects with mutant alleles of PPAR-gamma or uncoupling protein-1 [6,7]. In the long term, the intake of fermented doenjang improved glucose tolerance and inhibited hyperglycemia in high-fat diet-induced obese mice [8,9]. These health benefits may be due to the higher content of aglycone isoflavones and the diversity and abundance of *Bacillus* probiotic strains [4,9].

Despite the health benefits, doenjang fermentation requires a large amount of salt. Doenjang is traditionally made from fermented meju that has been dipped in a 18%–20% salt solution for more than 30 days [15]. Doenjang is fermented with 4 kg of meju dipped in brine, 10 L of water, and 1.25 kg of salt; the final salinity of traditional doenjang is 12% [2].

The main dietary sources of sodium in Korea are kimchi, salt, soy sauce, and soybean paste [16]. The World Health Organization recommends adults eat less than 2000 mg of sodium or 5 g of salt every day. Elevated sodium levels can raise blood pressure and may increase the risk of heart disease and stroke [17]. Thus, reducing sodium intake is recommended in the guidelines of many organizations. The optimal level of sodium intake is controversial; however, the sodium reduction program will help people prevent cardiovascular disease in communities that eat large amounts of salt [18]. However, although there are few studies that have combined doenjang and salt, there is a lack of research on mechanisms to prevent the side effects of salt. We hypothesized that consumption of traditional fermented foods would offset the side effects of table salt intake. Therefore, the antihypertensive effects of doenjang, a traditional fermented soybean paste, on the regulation of blood pressure were investigated in Sprague–Dawley (SD) rats.

## 2. Materials and Methods

### 2.1. Preparation of Doenjang

**Figure 1:**
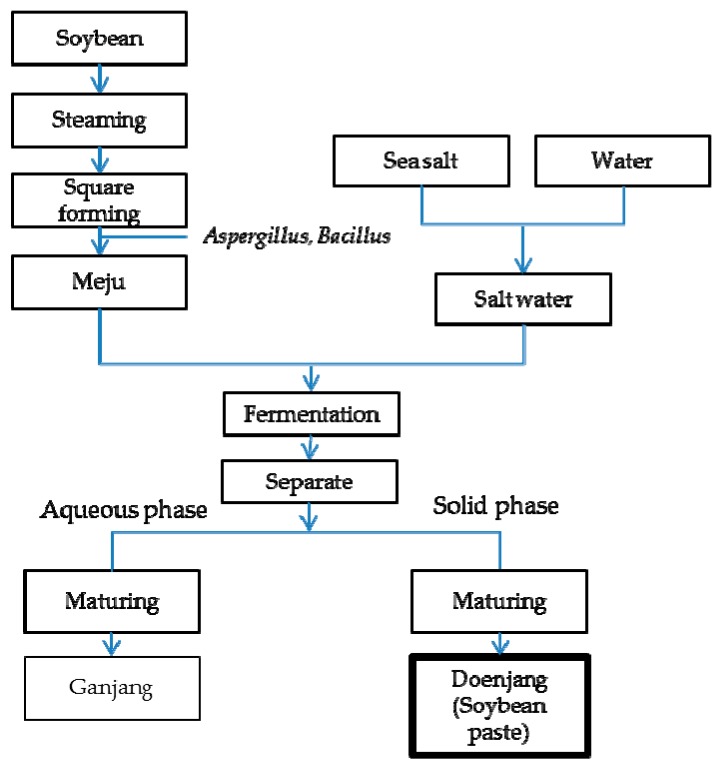
A manufacturing process diagram of doenjang. Meju, block of fermented soybeans; sea salt, manufacture of common salt by solar heat.

Doenjang was supplied by SunChangJangLye Co., Ltd. (Sunchang-gun, Korea). Meju, soybean fermented with *Aspergillus oryzae* and *Bacillus subtilis* for one month, and saltwater (26%, *w*/*v*) were mixed at a 1:3 ratio and fermented for 2 months. When the meju fermentation is complete, it is separated into the liquid phase and the solid phase. The crushed solid phase was matured for 6 months (Figure 1). Matured doenjang was stored by freeze drying. Freeze-dried doenjang and salt (Samchun Chemical, Seoul, Korea) were dissolved in distilled water to a salinity of 8% [19,20,21] using Mohr’s method [22].

### 2.2. Animal Study

Male SD rats, aged three weeks, were purchased from Central Lab Animal Inc. (Seoul, Korea). The animals were fed on an AIN-76 diet (standard diet, Research Diets, New Brunswick, NY, USA) for one week, and eighteen rats were randomly divided into three groups (*n* = 6): normal-salt group (0.3%, NS), high-salt group (8%, HS), and high-salt with doenjang group (HSD). Animals were kept at a temperature of 24 ± 2 °C with a humidity of 60 ± 5% and a light/dark cycle of 12:12 h. They were given free access to the AIN-76 diet and tap water.

The rats were fed orally administered 10 mL/kg body weight (BW) for the eight-week experimental period (Table 1). From the fifth to seventh weeks of the experimental period, rats were housed in metabolic cages for 24 h a week. Urine and feces were collected for analysis. The Animal and Use Committee of Chonbuk National University approved the experimental protocol (CBNU 2017-0016).

### 2.3. Collection of Serum and Organs

After 12 h of overnight fasting, rats were anesthetized with 2 mg/kg BW of alfaxan (Jurox, Australia) and 0.5 mL/kg BW of rompun (Bayer, Seoul, Korea) by intramuscular injection, and blood was collected by orbital vein puncture. Serum was centrifuged at 1100× *g* for 15 min at 4 °C. Liver and kidney were harvested, rinsed, and weighed. Both tissues and serum were stored at −80 °C until analysis.

### 2.4. Measurement of Blood Pressure

Blood pressure was assessed weekly by the tail-cuff method (BP-2000; Visitech Systems, Inc., Apex, NC, USA) after 6 h of oral administration.

### 2.5. Serum Profile Analysis

Serum glutamate oxaloacetate transaminase (GOT) and glutamate pyruvate transaminase (GPT) were analyzed using a commercially available kit (Asan Pharmaceutical Co., Seoul, Korea).

Renin, angiotensin II (Ang II), and aldosterone in serum were measured by ELISA using a Rat Renin ELISA Kit (MyBioSource, San Diego, CA, USA), Angiotensin II ELISA Kit, and Aldosterone ELISA Kit (Enzo Life Sciences, Inc., Farmingdale, NY, USA).

### 2.6. Ion Content Analysis in Feces and Urine

Sodium and potassium ion concentrations in feces and urine on the 7th week were analyzed by inductively coupled plasma-optical emission spectrometry (ICP-OES; Optima 8300DV, Perkin Elmer, Waltham, MA, USA) at Wonkwang University Wonnature (Iksan, Korea).

### 2.7. Gene Expression in the Kidney Cortex

Total RNA was extracted from the kidney cortex using Trizol reagent (Invitrogen Life Technologies, Carlsbad, CA, USA), and the concentration was measured with BioDrop (Biochrom, Holliston, MA, USA). The RNA was reversed to complementary DNA (cDNA) using a high-capacity cDNA reverse transcription kit (Applied Biosystems, Foster City, CA, USA). The expression levels were quantified with real-time PCR using SYBR Green PCR Master Mix (Toyobo, Osaka, Japan) and a 7500 Real-Time PCR system (Applied Biosystems). The relative gene expression levels were analyzed by the 2-ΔΔCt (-delta delta comparative threshold) method using β-actin as the reference gene. Primer sequences for the angiotensin II type 1 receptor (AT1 receptor, sense 5′-ACTCTTTCCTACCGCCCTTC-3′, antisense 5′-TTAGCCCAAATGGTCCTCTG-3′), angiotensin-converting enzyme (ACE, sense 5′-GAGCCATCCTTCCCTTTTTC-3′, antisense 5′-GGCTGCAGCTCCTGGTATAG-3′), mineralocorticoid receptor (MR, sense 5′-GCTTTGATGGTAGCTGCG-3′, antisense 5′-TGAGCACCAATCCGGTAG-3′), Na^+^/K^+^ ATPase alpha 1 (NKAα1, sense 5′-CCGGAATTCTGCCTTCCCCTACTCCCTTCTCATC-3′, antisense 5′-TGCTCTAGACTTCCCCGCTGTCGTCCCCGTCCAC-3′), Na^+^/H^+^ exchanger3 (NHE3, sense 5′-GGAACAGAGGCGGAGGAGCAT-3′, antisense 5′-GAAGTTGTGTGCCAGATTCT-3′), Na^+^/Ca^2+^ exchanger (NCX, sense 5′-GCGATTGCTTGTCTCGGGTC-3′, antisense 5′-CCACAGGTGTCCTCAAAGTCC-3′), Na^+^/HCO_3_^−^ co-exchanger (NBC, sense 5′-GGCACAGAGAGAGGAGGCTT-3′, antisense 5′-TGTCTTCCCAATGTCAGCCAG-3′) were used.

### 2.8. Analysis of Microbial Communities in Doenjang

Chunlab, Inc. (Seoul, Korea) performed the microbial community analysis of doenjang.

### 2.9. Statistical Analysis

The data were analyzed using one-way ANOVA with SPSS version 12.0 (SPSS Inc., Chicago, IL, USA). Values are expressed as mean ± standard deviation. The differences among groups were assessed using the Duncan’s multiple range tests. Statistical significance was considered at *p* < 0.05.

## 3. Results

### 3.1. Metabolic Characterization and Serum Chemistry

The initial BW, final BW and feed intake were not significantly different in all groups. The water intake and urine volume were increased in the HS group compared with the HSD group. Although salt intake was the same, water intake was decreased in the HSD group (Table 2).

Liver and kidney weights were not significantly different in all groups. In addition, liver-to-BW and kidney-to-BW ratios were no significant differences among groups. Significant difference was not observed in serum GOT and GPT levels among groups (Table 3).

### 3.2. Systolic Blood Pressure

**Figure 2:**
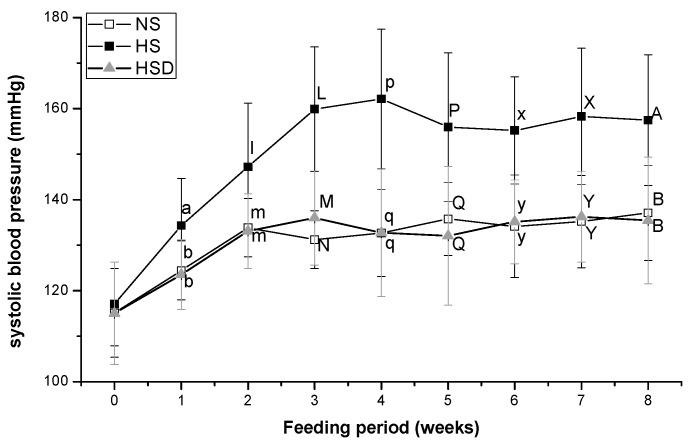
Changes in systolic blood pressure in Sprague–Dawley (SD) rats fed with high-salt diet. Values are the mean ± standard deviation, with different letters significantly different (*p* < 0.05) by Duncan’s multiple range test. Six rats were assigned to each group. NS normal-salt group; HS, high-salt group; HSD, high-salt with doenjang group.

Initial systolic blood pressure was the same in all three groups. From the first to eighth week of the feeding period, the systolic blood pressure in the HS group was higher than in the other groups. However, significant differences were not observed in systolic blood pressure between the NS and HSD groups at the end of the experiment (Figure 2). Final systolic blood pressure was 157.45 ± 14.36 mmHg in HS, 135.44 ± 13.91 mmHg in HSD, and 137.14 ± 10.44 mmHg in NS groups.

### 3.3. Ion Concentration in Feces and Urine

**Figure 3:**
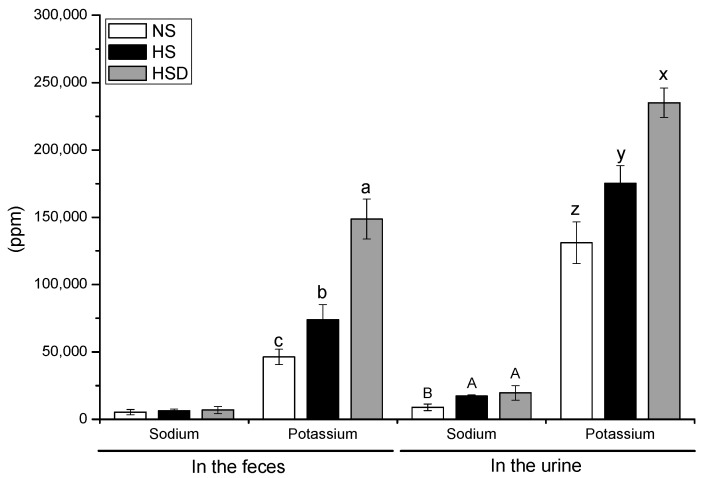
Ion concentrations in feces and urine in SD rats fed with high-salt diet. Values are the mean ± standard deviation, with different letters significantly different (*p* < 0.05) by Duncan’s multiple range test. Six rats were assigned to each group. NS, normal-salt group; HS, high-salt group; HSD, high-salt with doenjang group.

Sodium and potassium levels in feces and urine are shown in Figure 3. The sodium concentration in feces was not significant in three different groups. Fecal and urine potassium concentration was significantly higher in the HSD group than in the HS and NS groups. Sodium excretion in urine was significantly higher than NS in HS and HSD groups with a large amount of salt ingested.

### 3.4. Renin-Angiotensin-Aldosterone Levels in Serum

The serum renin-angiotensin-aldosterone (RAA) levels were analyzed (Table 4). Renin and aldosterone concentrations were slightly and significantly decreased in the HSD group compared to the HS group. Ang II level was significantly increased in the HS and HSD groups compared with the NS group.

### 3.5. Relative Gene Expression in the Kidney Cortex

**Figure 4:**
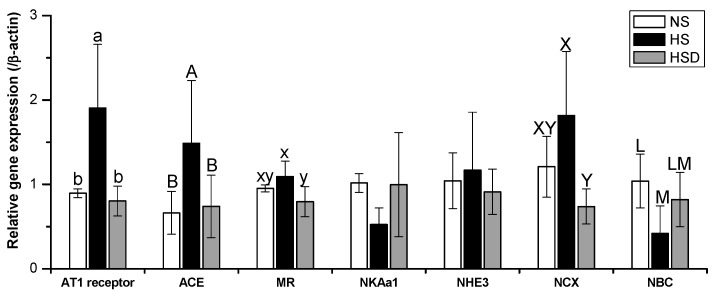
The mRNA expression of the kidney cortex in SD rats fed with high-salt diet. Values are the mean ± standard deviation, with different letters significantly different (*p* < 0.05) by Duncan’s multiple range test. Six rats were assigned to each group. NS, normal-salt group; HS, high-salt group; HSD, high-salt with doenjang group; AT1 receptor, angiotensin II type 1 receptor; ACE, angiotensin-converting enzyme; MR, mineralocorticoid receptor; NKAα1, Na^+^/K^+^ ATPase alpha 1; NHE3, Na^+/^H^+^ exchanger3; NCX, Na^+^/Ca^2+^ exchanger; NBC, Na^+/^HCO^3−^ co-exchanger.

The mRNA expression of the kidney cortex was analyzed (Figure 4). The mRNA expression of Ang II type 1 (AT1) receptor, Ang-converting enzyme (ACE), mineralocorticoid receptor (MR), and Na^+^/Ca^2+^ exchanger (NCX) was significantly higher in the HS group than in the HSD groups. Na^+^/HCO_3_^−^ co-exchanger (NBC) mRNA expression was significantly lower in the HS group than in the NS group. However, mRNA expression of the Na^+^/K^+^ ATPase α1 (NKAα1) and Na^+^/H^+^ exchanger 3 (NHE3) was not significantly in three different groups. Genetic changes in the HSD group were not significantly different from those in the NS group.

### 3.6. Microbial Community in Doenjang

**Figure 5:**
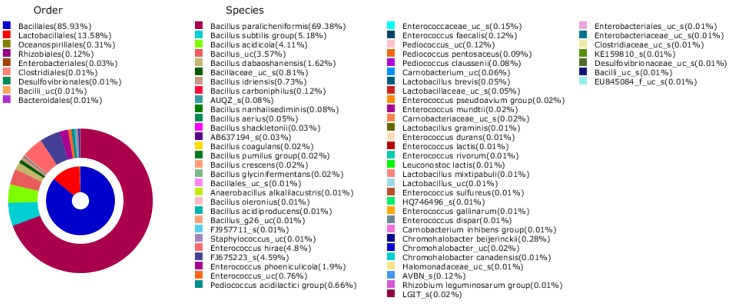
Microbial community in doenjang.

The microbial community in doenjang used in this experiment is shown in Figure 5 and consisted of the following: *Bacillales* (85.93%), *Lactobacillales* (13.58%), *Oceanospirillales* (0.31%), *Rhizobiales* (0.12%), *Enterobacteriales* (0.03%), *Clostridiales* (0.01%), *Desulfovibrionales* (0.01%), *Bacilli_uc* (0.01%), and *Bacteroidales* (0.01%). The most common species in doenjang were *Bacillus paralicheniformis* (69.38%), *Bacillus subtilis* group (5.18%), *Bacillus acidicola* (4.11%), *Bacillus_uc* (3.57%), and *Bacillus dabaoshanensis* (1.62%).

## 4. Discussion

Doenjang, a fermented soybean paste, is made from the fermentation of soybeans, salt, and water using a traditional method. The first step in making doenjang is to prepare meju by steeping, steaming, and forming the soybeans [10]. The second step is to soak fermented soybeans in saltwater and ferment them in natural conditions for one or two months [23]. The solid is separated from the liquid, which is collected to make the doenjang. The cooked cereals, salt, and crushed meju are added and left to ripen for 3–6 months [24]. Doenjang made from soybean and salt is traditionally used as both a condiment and has been used as a portion of health food that has anti-obesity, anti-diabetic, anti-cancer, and anti-inflammatory activities. Doenjang has ACE inhibitory effects that can help prevent increased blood pressure [24,25]. *Lactobacillus rhamnosus* in doenjang showed vigorous proteolytic activity and could aid in generating bioactive peptides [26].

In the present study, the intake of doenjang decreased blood pressure through modulation of the RAA system (RAAS), known to regulate blood pressure and fluid and electrolyte balance [27]. The RAAS plays a central role in regulating blood pressure by maintaining sodium and water homeostasis and vascular tone. Renin, as an inactive form, is synthesized from the kidney and released into the circulatory system in response to low levels of sodium in the tubular, low blood pressure in the arterioles of a renal glomerulus, and sympathetic activation. The active renin facilitates the division of angiotensinogen, which is cleaved by ACE to create Ang II, the main effector in the RAAS. The synthesis and secretion of aldosterone, another effector molecule in the RAAS, are stimulated by Ang II through the AT1 receptor in the adrenal cortex [28].

Although the rats in the HSD group consumed more calories due to the addition of doenjang, BW was not significantly different between the three groups. Liver weight, GOT, and GPT levels did not significantly between the groups; however, the liver/BW ratio was significantly lower in the HSD group than in the HS group. The liver weight in 2.3% NaCl group was significantly bigger than in commercial diet (0.3% NaCl) [29]. However, hepatic weight in the Japanese soybean paste (miso) diet group was decreased than in the NaCl diet group [29]. The initial stage of hypertension was not associated with overall renal failure, a small number of glomeruli, or glomerular hypertension [29]. Salt intake for eight weeks did not cause hepatic or renal injury, likely because of the short-term ingestion. The metabolism of doenjang is thought to be slower compared to the response to high-salt diet and decreases the risk of development and progression of hypertension and kidney damage.

Salt intake is an essential factor in controlling urination and water intake. Higher salt consumption increases urinary output and water intake to comparable levels. Increases in serum sodium and serum osmotic pressure stimulate thirst and antidiuretic hormones, causing increased fluid intake, decreased serum osmotic pressure, and increased urine volume [30].

A high level of sodium intake is reportedly associated with increased blood pressure. In the present study, the elevated blood pressure observed in rats fed a high-salt diet was consistent with previous studies [30,31]. Changes in systolic blood pressure were studied in spontaneously hypertensive rats fed a doenjang for nine weeks. Doenjang fermented with *Monascus* koji significantly decreased systolic blood pressure compared with a commercial diet [32]. Watanabe et al. showed that miso reduced blood pressure in SD and Dahl rats despite high salt content. Stroke-prone spontaneously hypertensive rats fed a miso diet showed decreased blood pressure and increased life survival compared with rats fed a high-salt diet containing 2.5% NaCl [33]. Ingestion of soybean paste, including doenjang and miso, is considered a significant source of dietary salt that does not increase blood pressure. Triple injections of His-His-Leu isolated peptides from doenjang lowered systolic blood pressure in spontaneously hypertensive rats [34]. In previous studies, doenjang extract exhibited ACE inhibitory activity in vitro [25]. When consuming fermented doenjang, dipeptides such as arginine-proline, which can produce an ACE inhibitory effect, are also ingested. Dipeptides and other peptide substances are known to prevent high blood pressure or have blood pressure depressant abilities [24].

The relationship between estimated potassium excretion and blood pressure is consistent with a systolic pressure of 0.65 mmHg per gram of potassium and a decrease in diastolic pressure of 0.42 mmHg per gram [35]. Sodium and potassium excretion, which is estimated as surrogate marker for ingestion, and blood pressure records in adults showed a non-linear correlation. If sodium excretion remains constant and potassium excretion is high, blood pressure decreases [35]. The sodium excreted in urine was not significantly different in the HS and HSD groups; however, the potassium excreted in urine was significantly elevated in the HSD group than in the HS group. The difference in ion excretion may also have contributed to lower blood pressure in rats in the HSD group.

In conventional theory, the typical reaction to the increase in salt intake is the suppression of circulating RAA hormones. Circulating renin activity and aldosterone concentrations were actively suppressed by a high-sodium diet [36]. Changes in arterial pressure were not observed with the high-sodium diet, although levels of Ang II and aldosterone were lessened in the 4% and 8% NaCl groups [19]. However, Wang et al. propose that renal RAAS action is independent or Versa of plasma RAA level at high salt intake, and improper activation of the RAAS in the kidney may contribute directly to hypertension and kidney damage [37]. Although the high-salt diet did not inhibit RAA level, doenjang lowered serum levels of renin and aldosterone in the present study.

A high-salt diet increased the mRNA expression of RAAS components in the kidney cortex. AT1 receptor and ACE mRNA expression in the kidney cortex was higher in the high-salt diet than in normal-salt diet groups. The RAAS in the kidney was inappropriately enhanced by high salt intake in SD rats [37]. High salt intake exacerbated blood pressure elevation during the development of hypertension in spontaneously hypertensive rats [38]. The increased salt content in the diet stimulates glomerular oxidative stress, which leads to the AT1 receptor, MR, and ACE up-regulation, subsequently causing hyperbole in sodium transporters, and providing to sodium retention and hypertension [39,40]. A high-salt diet could cause kidney damage through oxidative stress and modulation in the ACE/ACE2 ratio [41].

Electrogenic cationic pumps, as well as NKA α1, NCX, NBC, and NHE3, are essential for transcellular movement of water and ions in osmoregulatory epithelia. Increasing Ang II concentration decreased NKA activity in eel enterocytes; however, NKA activity was not affected by concentration of Ang II in saline adaptation [42]. The abundance of NHE3 and NKAα1 in the kidney cortex and medulla was not changed in SD rats fed a 4% NaCl diet [43]. A chronic high-salt diet increased renal NCX1 mRNA expression in Wistar–Kyoto rats [44].

Regarding the regulation of the sodium–potassium pump, Ang II activates PKCβ at low concentrations. As a result, the NKAα1 subunit is phosphorylated, and NKA is incorporated into the plasma membrane. Aldosterone is reported to stimulate the migration of the NKAα1 subunit from the intracellular compartment to the basal side membrane surface of the distal nephron. NBC-1 plays a vital role in regulating renal acid-alkaline balance, maintaining pH in the blood and the cells, and regulating sodium transport through NHE-3 in the proximal tubule. The regulations of NHE3 and NBC-1 have standard features due to the interrelated functions of the two ion transporters. The isolation of individual levels in function regulation is severe because many intracellular factors are involved in modulating the transporter function at different levels and may act as universal regulators of several transporters [45].

In this study, we confirmed that doenjang affects the RAA mechanism. However, doenjang containing high salt does not have a direct effect on blood pressure increase in a healthy animal model. The results from the present study indicate the importance of the microbial community in doenjang. The majority of microorganisms in doenjang are *Bacillus paralicheniformis*, which is tolerant to 10% NaCl [46]. Furthermore, probiotic microorganisms, including *Bacillus* strains, may have exerted health benefits. The consumption of probiotics may improve blood pressure control. Probiotics can be used as a potential supplement for future interventions to prevent high blood pressure or improve blood pressure control [47]. Although the mechanism is different from that of the RAAS, probiotics are expected to contribute to blood pressure control.

The primary sources of dietary sodium for the Korean people are kimchi, salt, soy sauce, and soybean paste [16]. Because doenjang significantly contributes to salt intake in Koreans, the focus in the present study was on the effects of salt contained in doenjang on blood pressure. The consumption of a large amount of traditional, salty fermented food was not found to be a direct cause of hypertension. For example, the high consumption of kimchi was not associated with an increased risk of hypertension in Korean adults [48]. The results from the present study showed that the intake of doenjang in normal healthy animals might improve blood pressure.

## 5. Conclusions

Our study demonstrated that doenjang with high-salt content improved blood pressure in SD rats. The intake of doenjang increased potassium excretion in feces and urine compared to high salt intake. Furthermore, doenjang decreased renin and aldosterone levels in serum and the expression of the AT1 receptor, ACE, and MR in the kidney cortex compared to high salt intake. These results indicate that eating traditional salty fermented food is not a direct cause of hypertension, and the intake of doenjang in normal healthy animals improved blood pressure.

## Figures and Tables

**Table 1:** Ingredients administered orally during the experimental period.

Group ^1^	Energy (kcal)	Ash (g)	Crud Protein (g)	Carbohydrate (g)	Crud Fat (g)	Salt (%)	Sodium (mg/kg BW/day)	Potassium (mg/kg BW/day)
NS	0	0	0	0	0	0	0	0
HS	0	0	0	0	0	8.00	314.72	0
HSD	72.75	7.37	4.80	4.24	4.06	8.02	229.29	14.98

Body weight (BW). ^1^ The normal-salt (NS) group was fed with distilled water. The high-salt (HS) group was fed with saltwater that dissolved NaCl in distilled water. The high-salt with doenjang (HSD) group was fed with doenjang solution that mixed freeze-dried deonjang in distilled water.

**Table 2:** Metabolic characterization in Sprague–Dawley (SD) rats fed with high-salt diet.

Group ^1^	Initial Body Weight (g)	Final Body Weight (g)	Diet Intake (g/day)	Water Intake (mL/day)	Urine Volume (mL/day)
NS	128.24 ± 8.27	358.86 ± 33.18	16.97 ± 2.97	22.65 ± 4.81 ^a,b^	9.54 ± 3.24 ^c^
HS	129.40 ± 7.28	359.73 ± 26.51	19.35 ± 5.61	25.96 ± 6.4 ^a^	16.17 ± 3.02 ^a^
HSD	129.51 ± 9.03	356.09 ± 47.93	18.03 ± 3.19	18.88 ± 4.53 ^b^	13.25 ± 2.53 ^b^

Values are the mean ± standard deviation, with different letters significantly different (*p* < 0.05) by Duncan’s multiple range test. Six rats were assigned to each group. ^1^ NS, normal-salt group; HS, high-salt group; HSD, high-salt with doenjang group.

**Table 3:** Organ weights and serum chemistry in SD rats fed with high-salt diet.

Group ^1^	Liver Weight (g)	Kidney Weight (g)	Liver/BW	Kidney/BW	GOT (IU/L)	GPT (IU/L)
NS	11.55 ± 1.01	2.65 ± 0.34	3.23 ± 0.19 ^b^	0.74 ± 0.06	25.33 ± 9	2.62 ± 2.35
HS	12.7 ± 1.13	2.81 ± 0.24	3.53 ± 0.18 ^a^	0.78 ± 0.06	24.75 ± 4.81	2.31 ± 0.77
HSD	11.88 ± 2.06	2.7 ± 0.37	3.33 ± 0.26 ^b^	0.76 ± 0.08	26.53 ± 4.88	2.31 ± 2.04

Values are the mean ± standard deviation, with different letters significantly different (*p* < 0.05) by Duncan’s multiple range test. Six rats were assigned to each group. ^1^ NS, normal-salt group; HS, high-salt group; HSD, high-salt with doenjang group; BW, body weight; GOT, Glutamate oxaloacetate transaminase; GPT, glutamate pyruvate transaminase.

**Table 4:** Renin-angiotensin-aldosterone (RAA) levels in serum (pg/mL).

Group ^1^	Renin	Angiotensin II	Aldosterone
NS	41.72± 1.77 ^a^	109.29 ± 5.19 ^b^	19.88 ± 1.10 ^a^^,^^b^
HS	43.10± 3.36 ^a^	121.60 ± 4.98 ^a^	21.37 ± 0.97 ^a^
HSD	38.22± 2.66 ^b^	118.85 ± 8.01 ^a^	19.23 ± 2.03 ^b^

Values are the mean ± standard deviation, with different letters significantly different (*p* < 0.05) by Duncan’s multiple range test. Six rats were assigned to each group. ^1^ NS, normal-salt group; HS, high-salt group; HSD, high-salt with doenjang group; RAA, renin-angiotensin-aldosterone.
